# What lies in the year ahead for medical education? A medical student’s perspective during the COVID-19 pandemic

**DOI:** 10.1080/10872981.2020.1781749

**Published:** 2020-06-16

**Authors:** Olivia Raymond-Hayling

**Affiliations:** College of Medical and Dental Sciences, University of Birmingham, Birmingham, UK

**Keywords:** COVID-19, clinical education, online teaching, physical examination, social distancing

Dear Editor,

2020 has provided more unpredictability than anyone could have foreseen. For both healthcare and education there a huge amount of uncertainty, for the coming year, months and even weeks. Many of us have been questioning the future of medical education across the world whilst the COVID-19 crisis continues. Institutions have announced movements to online methods of teaching and learning, reduced face-to-face contact and have considered re-evaluation of current methods of examination. [1,2] At this point, whether this applies to healthcare education is still not known. [3]

What does all this mean for medical students? Is it possible to provide a satisfactory medical education with the view of minimal in-person teaching? A cohort of doctors is needed each time the academic year comes to a close; delaying the qualification of a cohort of doctors is not an option. Nevertheless, putting public and patient safety at risk goes against all ethical principles of medicine. [4]

There is definitely scope for online teaching during pre-medical/pre-clinical education. Students are often taught in lecture and tutorial formats which can be quite simply delivered online, if the appropriate training is given to teachers. In addition to this, developing technology has increased the popularity of virtual anatomy models to be used. [5] This would reduce the need for close contact in dissection rooms. Written exams are also already currently being carried out online. Despite all these promising adjustments, a huge area that would be compromised is communication. Developing the interpersonal skills and confidence to speak to patients, discuss patient care with colleagues and present academic work, is a key skill that needs to be employed from the beginning of medical school. Even though there is good prospect for interactivity through video teaching in pre-clinical education, there would undoubtedly be a shortfall in this area.

For students in clinical phases, altering teaching and examining methods is not quite as simple. Although roleplay, examination and clinical procedures in video formats are widely used as a tool for observance, it comes with a more structured format than in real clinical care. [6] Using real patient notes, talking to staff involved in their care and deciphering their journey into hospital is not something that can be replicated. Clinical placement may have to involve two-metre distancing where students are observing, and personal protective equipment where close contact is required.

Many medical school exams, for instance the Objective Structured Clinical Examinations (OSCE) format, include voluntary examiners, patients and actors. [6] The patients are subjected to students in succession speaking in close proximity, as well as demonstrating medical examinations including physical contact. Since these participants are taking part for a hypothetical scenario, one could say that they are being put at unnecessary risk of illness. History-taking could well be practised and examined over video communication, which may have to happen this coming academic year 2020/21.

There are some forms of communication that involve remote contact regardless; perhaps by shifting from the focus of face-to-face communication, these other areas could be assessed. Hypothetically, if the original format of an assessment was to present a patient’s given history and examination to a senior, this could be modified to referring a patient to a specialist over the phone, as is often done in a hospital. This would still allow a student to practice summarising a patient’s presentation yet removing close contact to an examiner at the same time. How these remote examinations could be invigilated is another question yet to be answered.

The single area that clearly has very little room for adaptation is assessment of physical examination, and other than using unrealistic simulation models, there is no way to get around this. Extra measures may have to be taken to ensure that risk of infection is minimised, similar to those mentioned for clinical placements above.

Alternatively, a move from OSCE format to in-hospital assessment of history and examination. Such formats are used for doctors in their stages of training, one of which is the mini clinical evaluation exercise (mini-CEX). [7] This could ensure that a patient is only encountered once by a student, as opposed to the OSCE format where they may be encountered far more times.

Overall, there are many aspects of medical education can be adapted for this coming year; nevertheless it may be inevitable that the next cohort of doctors have less experience than is stated in their curriculum. The impact of this, if any, will be observed in the next cohort of graduating medical students.
